# Airway Serous Cells: A Comparative Study of Spatial Distribution and Abundance among Species

**DOI:** 10.35534/jrbtm.2024.10013

**Published:** 2024-08-07

**Authors:** Yuanpu Peter Di, Hongmei Mou

**Affiliations:** 1 Department of Environmental and Occupational Health, University of Pittsburgh, Pittsburgh, PA 15261, USA; 2 Mucosal Immunology and Biology Research Center, Massachusetts General Hospital, Boston, MA 02114, USA

**Keywords:** Airway Serous Cells, Submucosal Glands, Surface Epithelial cells, BPIFA1, Humans, Pigs, Rodents

## Abstract

The conducting airways of the respiratory system play a crucial role in filtering, humidifying, and directing air into the lungs. Among the specialized cell types within these airways, airway serous cells are notable for their secretion of watery, protein-rich fluids and enzymes, which contribute to maintaining airway surface liquid homeostasis and defending against pathogens. However, the distribution and abundance of serous cells across different species in the conducting airways remain poorly understood. In this study, we addressed this gap by investigating the spatial distribution of the airway serous cell-specific marker BPI fold containing family A member 1 (BPIFA1) in humans, pigs, and mice. Our findings demonstrate significant variations in the distribution and abundance of serous cells among these species, potentially reflecting their different respiratory anatomy and evolutionary adaptations to diverse environmental challenges and respiratory demands. In humans and pigs, airway serous cells are predominantly found in the submucosal glands of the trachea and segmental bronchi, frequently overlapping with lysozyme-positive secretory cells. In contrast, rodents like mice exhibit a distinct pattern where serous cells are scarce in submucosal glands. Instead, rodent serous cells are primarily located at the epithelial surface from the trachea to the main bronchi, where many co-express the Club cell-specific protein SCGB1A1. The abundance of serous cells diminishes progressively in the intrapulmonary airways. Given that rodent models are widely utilized in respiratory research, understanding anatomical and cellular differences in airway serous cells is critical for interpreting experimental outcomes and translating findings to human respiratory diseases and therapeutic strategies. This comparative analysis enhances our understanding of airway biology across species and informs the selection and interpretation of animal models in respiratory studies.

## 1. Introduction

Airway serous cells are a distinct type of epithelial cell found predominantly in the conducting airways of mammals, including humans, pigs, and rodents. Serous cells are the primary defensive cells of the mucosa because they discharge antimicrobial compounds that deal efficiently with pathogens [1]. One of their primary roles is the secretion of water, antimicrobials, enzymes, and other components that contribute to maintaining mucosal immunity and airway homeostasis. Airway serous cells contribute approximately 10% of all proteins in the airway surface liquid (ASL) in the respiratory tract [2]. Their secretion is crucial for maintaining the hydration and integrity of the airway epithelium, supporting efficient mucociliary clearance, and providing a barrier against pathogens and environmental insults. Airway serous cells were first identified in the early 20th century through histological studies highlighting their distinct morphology and secretory functions. Early researchers used light microscopy to observe the granule-rich cytoplasm of these cells, distinguishing them from mucous cells [3–6]. The advent of electron microscopy in the mid-20th century allowed for more detailed visualization of the ultrastructural features of serous cells, including their homogeneous and electron-dense secretory granules [3,7–10]. Immunohistochemistry and molecular biology techniques in the latter half of the 20th century further characterized these cells. Markers such as lysozyme, lactoferrin, and BPIFA1 (short palate lung and nasal epithelial clone 1 (SPLUNC1)) became essential for identifying serous cells in tissue sections. These markers not only highlighted the presence of serous cells but also provided insights into their functional roles in antimicrobial defense and mucosal immunity. Recently, single-cell RNA sequencing (scRNA-seq) has revolutionized our understanding of cellular heterogeneity and function within complex tissues. Its application to the study of the cellular and molecular architecture of pig submucosal glands has yielded significant discoveries about the roles, distribution, and responses of different cell types in the respiratory system. Several key markers, including BPIFA1, SCGB1A1, DMBT1, OBP2B, and lysozyme, have been identified in sub-clusters of serous cells [11]. These markers provide a molecular basis for differentiating acinar serous cells from ductal serous cells as well as distinguishing serous cells from mucous and ciliated cells [11]. Among them, BPIFA1 is expressed exclusively in airway tissues and, therefore, is regarded as a unique marker to delineate airway serous cells.

BPIFA1 is a member of the BPI fold-containing (BPIF) protein family and is central to the function of airway serous cells. We originally characterized BPIFA1 as an ASL protein secreted apically in primary human airway epithelial cell cultures [12], the abundance of which was changed in multiple chronic lung diseases [13–16]. Importantly, we demonstrated that *BPIFA1* mRNA and protein were restricted to human serous cells of secretory ducts and submucosal glands [12]. BPIFA1 is notable for its antimicrobial properties, acting as a host defense molecule by binding to and neutralizing pathogens, thereby protecting the airway epithelium from infection [17–19]. Beyond its antimicrobial role, BPIFA1 contributes to immune modulation and regulation of inflammation within the airways [18]. Studies have shown that BPIFA1 can interact with immune cells and modulate cytokine production, influencing the local immune response and promoting airway homeostasis [18]. Furthermore, BPIFA1 can lower the surface tension of airway secretion with an activity similar to what surfactant proteins do in the alveolar space [17,20]. The expression and function of BPIFA1 and airway serous cells are tightly regulated under normal physiological conditions. However, their dysregulation has been implicated in various respiratory diseases. For instance, alterations in BPIFA1 expression or dysfunction of serous cells can compromise the protective barrier of the airway epithelium, leading to increased susceptibility to respiratory infections such as pneumonia and chronic obstructive pulmonary disease (COPD). Inflammatory conditions and exposure to environmental pollutants can also disrupt serous cell function, affecting ASL composition and impairing mucociliary clearance mechanisms. Understanding the roles of BPIFA1 and airway serous cells is crucial for developing novel therapeutic strategies targeting respiratory diseases. Research efforts are focused on elucidating the mechanisms underlying their regulation, exploring their interactions with pathogens and immune cells, and investigating their potential as biomarkers for respiratory health and disease progression. Moreover, advances in genetic and molecular techniques have enabled researchers to manipulate BPIFA1 expression in animal models, offering insights into its therapeutic potential in enhancing airway defense mechanisms.

## 2. Experimental Procedures

### 2.1. Airway Tissues, Immunohistochemistry, and Immunofluorescence

Healthy human bronchial tissues were from the discarded and rejected lungs that are not suitable for lung transplantation. The use of human de-identified tissues for research is under an IRB-approved protocol (#2017P001479, MGH). Mouse tracheal and lung tissues were from C57B6 mice at various ages (JAX 000664). Pig tracheal tissues were isolated from wild-type piglets at 3–4 months old (these were obtained as discarded tissues from the MGH surgical department). Human bronchial airways and animal tracheal tissues were fixed with 4% paraformaldehyde (PFA) at 4 °C overnight (>12 h). Afterward, the tissues were incubated in 30% sucrose in PBS at 4 °C for one day and then soaked in Tissue-Tek^®^ O.C.T. Compound for >12 h and then frozen in OCT for cryosectioning at 7 μm thickness. Primary antibodies utilized in this study were as follows: CCSP (EMD Millipore, Burlington, MA, USA, ABS1673, Goat polyclonal, dilution 1:250), SPLUNC1 (Lab-made antibodies, Rabbit polyclonal, dilution 1:1000). Antibodies specific for human BPIFA1 antibodies were generated using recombinant human BPIFA1-Gln20-Val256, while the antibodies specific for mouse BPIFA1 were generated using recombinant mouse BPIFA1-Gln20-Asn110), Lysozyme (Dako, Santa Clara, CA, USA, Rabbit polyclonal, A0099, dilution 1:200), For secondary labeling, antibodies conjugated with Alexa Fluor (488 and 594) were obtained from Life Technologies and used at a dilution of 1:500. The staining images were visualized and captured using an Olympus Fluoview FV10i Confocal Microscope or a Nikon A1 Confocal Laser microscope.

### 2.2. Quantification and Statistical Analysis

The staining quantification was performed by counting at least 3 sections and 5–10 random fields of view for each section with a 20× objective. The total number of cells was measured based on DAPI labeling. In some experiments, the quantification of differentiation was performed by calculating total lineage staining positivity per the total area. Data were presented as means with standard deviations of measurements unless stated otherwise. N ≥ 3. Statistical differences between samples were assessed with a Student two-tailed T-test. *p*-values below 0.05 were considered significant (**** *p* ≤ 0.0001, *** *p* ≤ 0.001, ** *p* ≤ 0.01, * *p* ≤ 0.05).

## 3. Results

### 3.1. BPIFA1^+^ Serous Cells Are Mainly Identified in the Submucosal Glands of the Large Airways of Healthy Humans and Pigs

**Figure 1.**
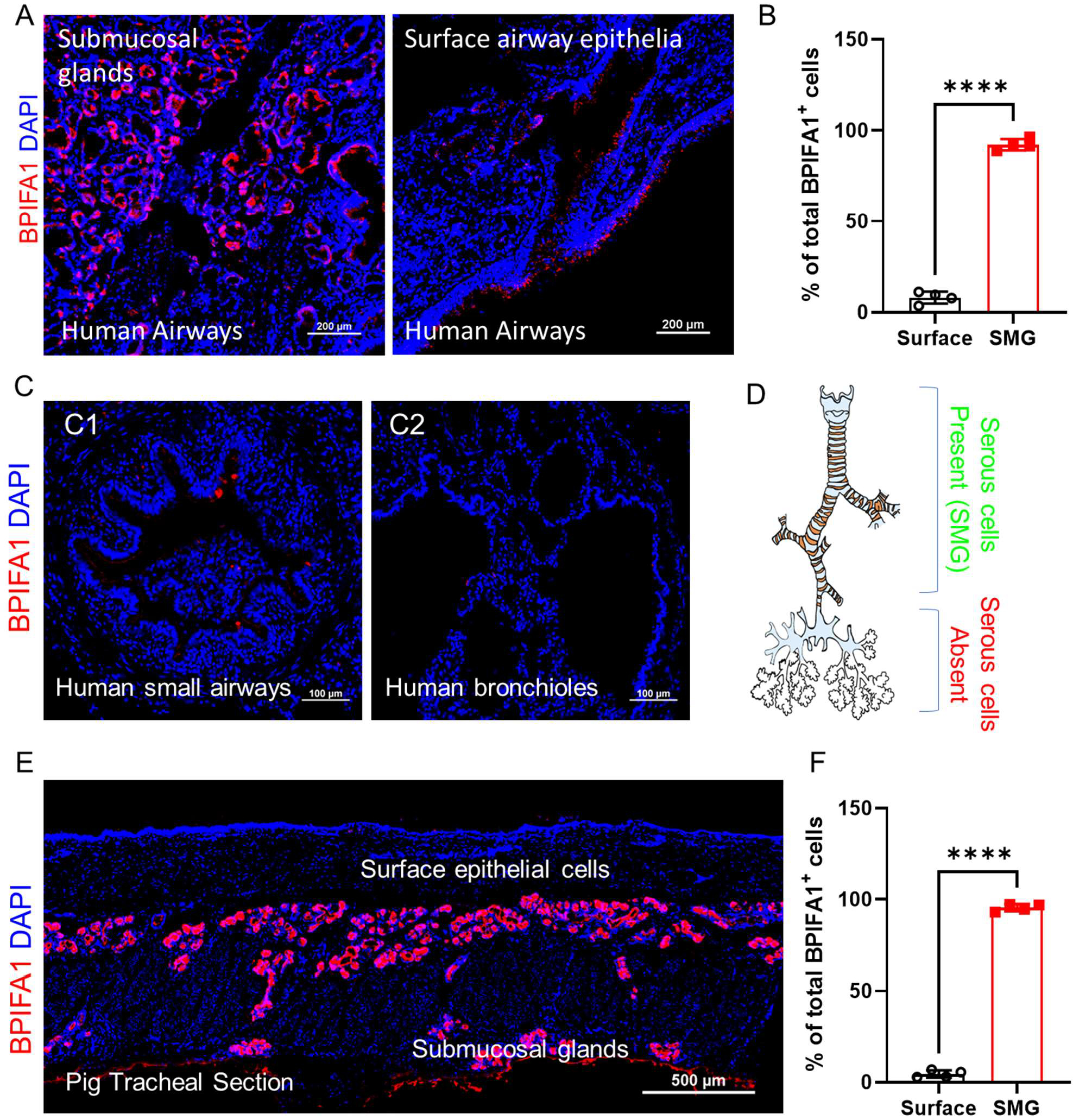
BPIFA1^+^ serous cells are mainly identified in the submucosal glands of the large airways of healthy humans and pigs. (**A**). Representative staining of BPIFA1^+^ airway serous cells on human bronchial sections (left panel, submucosal glands; right panel: surface airway epithelia). Scale bar: 200 μm. (**B**). Quantification of the percentage of BPIFA1^+^ airway serous cells that are identified in submucosal glands vs. at the airway surface on human bronchial sections. mean ± SEM, **** *p* < 0.0001. *n* = 4 independent tissues/sections. (**C**). Representative BPIFA1^+^ airway serous cells staining on human small airways (**C1**) and terminal bronchioles (**C2**). Scale bar: 100 μm. (**D**). Diagrammatic image illustrating the distribution of BPIFA1^+^ airway serous cells along the human conducting airways. BPIFA1^+^ serous cells are predominantly located in the submucosal glands of the trachea, bronchi, and segmental bronchi. These cells are absent in distal airways that lack submucosal glands and are not found in the lung parenchyma. (**E**). Representative staining of BPIFA1^+^ airway serous cells on pig tracheal sections. Scale bar: 500 μm. (**F**). Quantification of the percentage of BPIFA1^+^ airway serous cells that are identified in submucosal glands vs. at the airway surface on pig tracheal sections. mean ± SEM, **** *p* < 0.0001. *n* = 4 independent tissues/sections.

To investigate the distribution and abundance of serous cells in humans, we determined the expression of BPIFA1 utilizing specific anti-BPIFA1 antibody staining on the conducting airways of healthy individuals devoid of any respiratory or lung diseases to minimize potential alterations in serous cell numbers and BPIFA1 expression associated with disease states. Our staining results revealed that BPIFA1-positive cells are predominantly localized within the submucosal glands of large conducting airways, with occasional BPIFA1-positive cells sparsely distributed in the surface epithelium (Figure 1A,B). Submucosal glands in human airways decrease in number as they extend distally, and they are absent in smaller airways and bronchioles. Consistently, BPIFA1-positive cells are not detected in these regions (Figure 1C and summarized in Figure 1D). Although single-cell RNA sequencing in some studies identified small or trace amounts of BPIFA1 mRNA in basal cells, goblet cells, and alveolar epithelium, we did not detect BPIFA1 antibody immunopositivity in these cells (data not shown). Staining of BPIFA1 on pig airways exhibited a distribution pattern comparable to that in humans. Notably, BPIFA1-positive cells in pigs were exclusively confined to the submucosal glands, with no BPIFA1-positive cells detected on the surface epithelium (Figure 1E,F). BPIFA1 is responsive to environmental challenges that may affect their expression patterns. This discrepancy could potentially stem from the fact that experimental pigs were raised in controlled and clean environments, whereas human subjects had been exposed to various pathogens and respiratory viruses throughout their lives, despite not being clinically diagnosed with lung diseases.

### 3.2. BPIFA1^+^ and Lysozyme^+^ Serous Cells Have Distinct Distribution and Abundance in Human Airway Submucosal Glands

**Figure 2.**
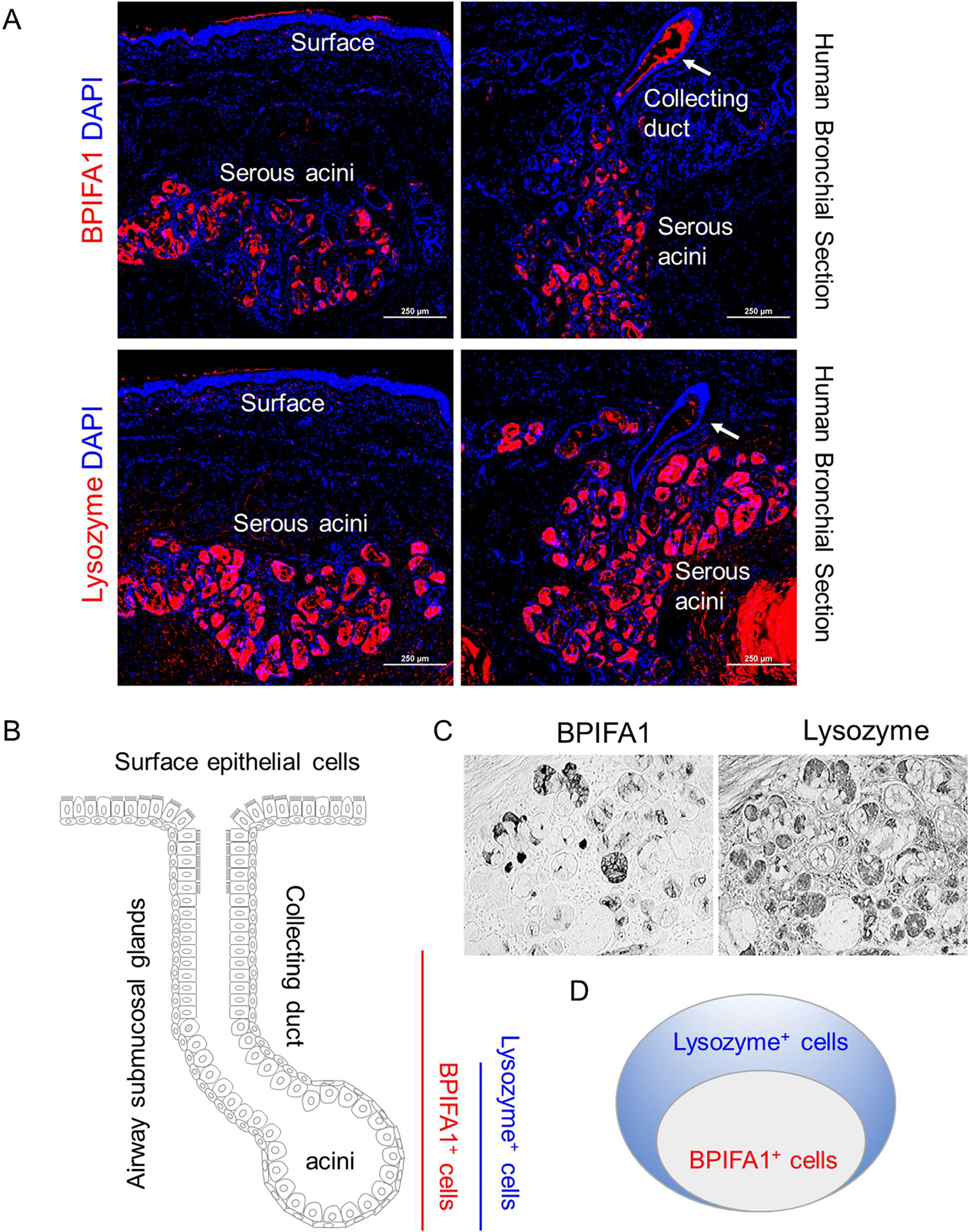
BPIFA1^+^ and Lysozyme^+^ serous cells have distinct distribution and abundance in human airway submucosal glands. (**A**). Representative staining of BPIFA1 and Lysozyme on human bronchial serial sections. Scale bar: 250 μm. (**B**). Diagrammatic image illustrating the distinct distribution of BPIFA1^+^ serous cells and Lysozyme^+^ serous cells in human airway submucosal glands. BPIFA1^+^ serous cells can be identified in both collecting ducts (indicated by arrow) and serous acini, whereas Lysozyme^+^ serous cells are restricted in serous acini. (**C**). Representative staining of BPIFA1 and lysozyme on serial sections of submucosal serous acini shows the presence of lysozyme-positive but BPIFA1-negative cells. Scale bar: 200 μm. (**D**). Diagrammatic image illustrating that BPIFA1-expressing serous cells are a subpopulation of lysozyme-expressing serous cells in the secretory acini of submucosal glands.

Submucosal glands consist of a tubular structure with secretory acini that produce mucins, enzymes, and other components, as well as collecting ducts that open onto the airway epithelial surface. Staining for BPIFA1 indicated that BPIFA1 immunopositivity can be identified in both the secretory acini and the collecting ducts (Figure 2A and summarized in Figure 2B). Interestingly, lysozyme staining revealed that this protein is restricted to the secretory acini of the submucosal glands and is absent in the epithelial cells lining the collecting ducts (Figure 2A,B). Furthermore, in the secretory acini, lysozyme immunopositivity is more widespread than that of BPIFA1 (Figure 2A). Staining on serial tissue sections suggested that there are many lysozyme-positive but BPIFA1-negative cells (Figure 2C). Therefore, BPIFA1-expressing serous cells might be a subpopulation of lysozyme-expressing serous cells in the secretory acini of submucosal glands (Figure 2D). The distinct physiological functions of these serous cell subpopulations are currently unknown and warrant further investigation.

### 3.3. Mouse BPIFA1^+^ Serous Cells Are Predominantly Present in the Surface Epithelial Cells of the Trachea and Main Bronchi

**Figure 3.**
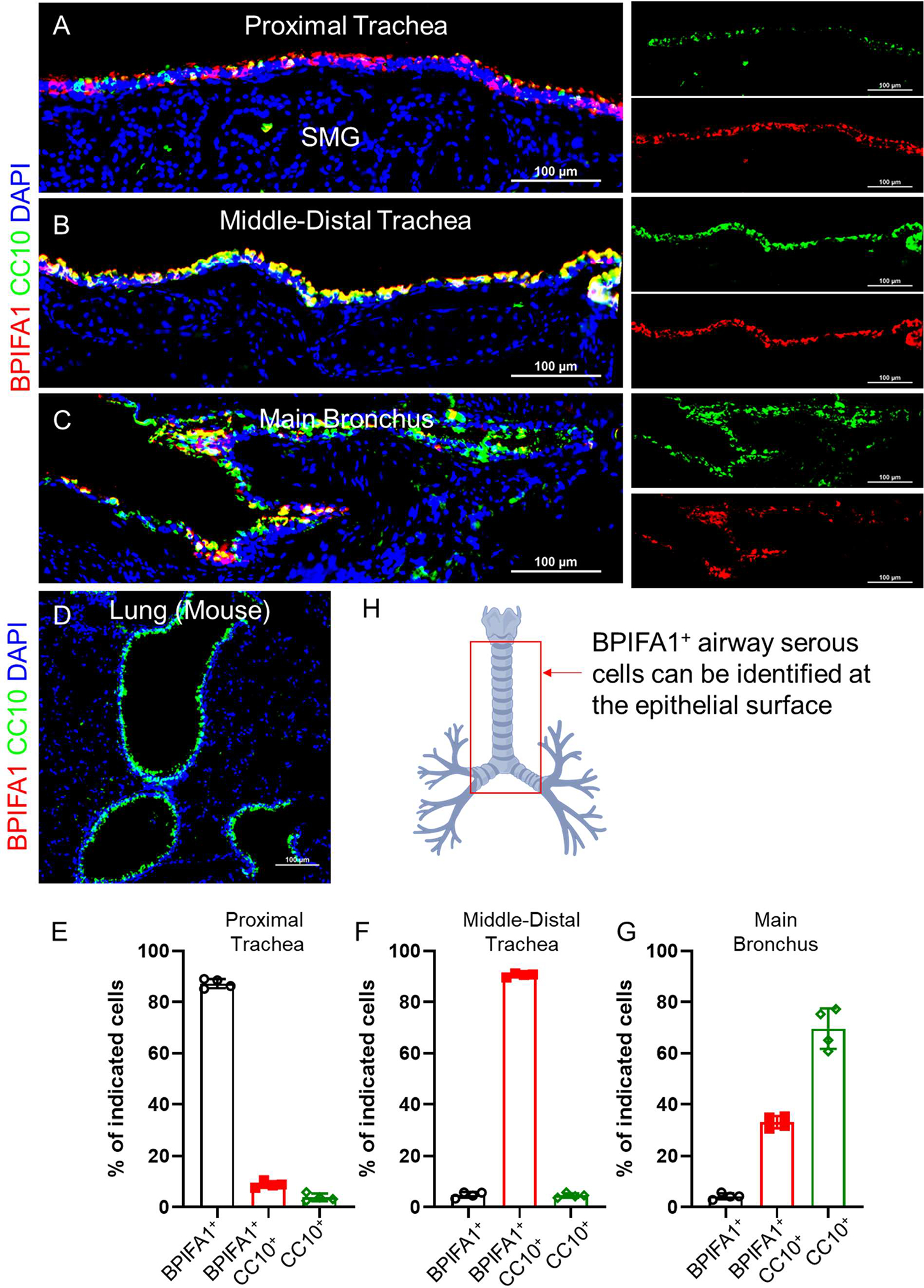
Mouse BPIFA1^+^ serous cells are predominantly present in the surface epithelial cells of the trachea and main bronchi. (**A**–**D**). Representative staining of BPIFA1 and CC10 on mouse proximal (**A**) and middle-distal tracheal sections (**B**), main bronchial sections (**C**), and lung sections (**D**). The yellow color indicates the co-expression of BPIFA1 (red) and CC10 (green). Scale bar: 100 μm. (**E**–**G**). Quantification of the percentage of BPIFA1^+^ (single positive), BPIFA1^+^CC10^+^ (double positive), and CC10^+^ (single positive) out of total airway epithelial cells that are either BPIFA1 positive or CC10 positive airway epithelial cells at proximal tracheal regions (**E**), and middle-distal tracheal regions (**F**), and bronchial regions (**G**). mean ± SEM, *n* = 4 independent tissues/sections. (**H**). Diagrammatic image illustrating the distribution of BPIFA1^+^ airway serous cells along the mouse conducting airways. BPIFA1^+^ serous cells are predominantly located in the epithelial surface of the trachea and extrapulmonary bronchi. BPIFA1^+^ airway serous cells are absent in intrapulmonary airways in the lungs.

Mouse submucosal glands are less prominent and sparse compared to those in humans and are primarily found in the proximal trachea. Additionally, these glands are fewer in number and smaller in size than those in humans, with a simpler structure characterized by fewer acini and smaller ducts. Intriguingly, when we stained BPIFA1 on mouse airways, we noticed that mouse serous cells were absent in submucosal glands (Figure 3A). However, a few BPIFA1-positive cells could occasionally be identified in glandular ductal epithelial cells. Mouse BPIFA1-positive serous cells are exclusively located in surface epithelial cells extending from the proximal trachea to the main bronchus (Figure 3A–C). Co-staining with the secretory cell lineage marker CC10 (Scgb1a1) with BPIFA1 indicated that these two proteins are co-expressed, particularly in the middle-distal tracheal regions (Figure 3B,F). In the proximal tracheal region, where there are fewer secretory Club cells, BPIFA1-expressing cells are more abundant than Club cells, and many BPIFA1-positive serous cells are negative for CC10 expression (Figure 3A,E). In contrast, in the main bronchus, where the Club cells are enriched, many CC10-positive cells are absent for BPIFA1 expression (Figure 3C,G). BPIFA1-positive cells gradually diminish as the bronchi extend distally, with no BPIFA1-positive cells identified in the intrapulmonary airways. The distribution of BPIFA1 serous cells in mouse conducting airways is summarized in Figure 3H.

### 3.4. BPIFA1^+^ Airway Serous Cells in the Mouse Trachea Decrease in Abundance with Aging

**Figure 4.**
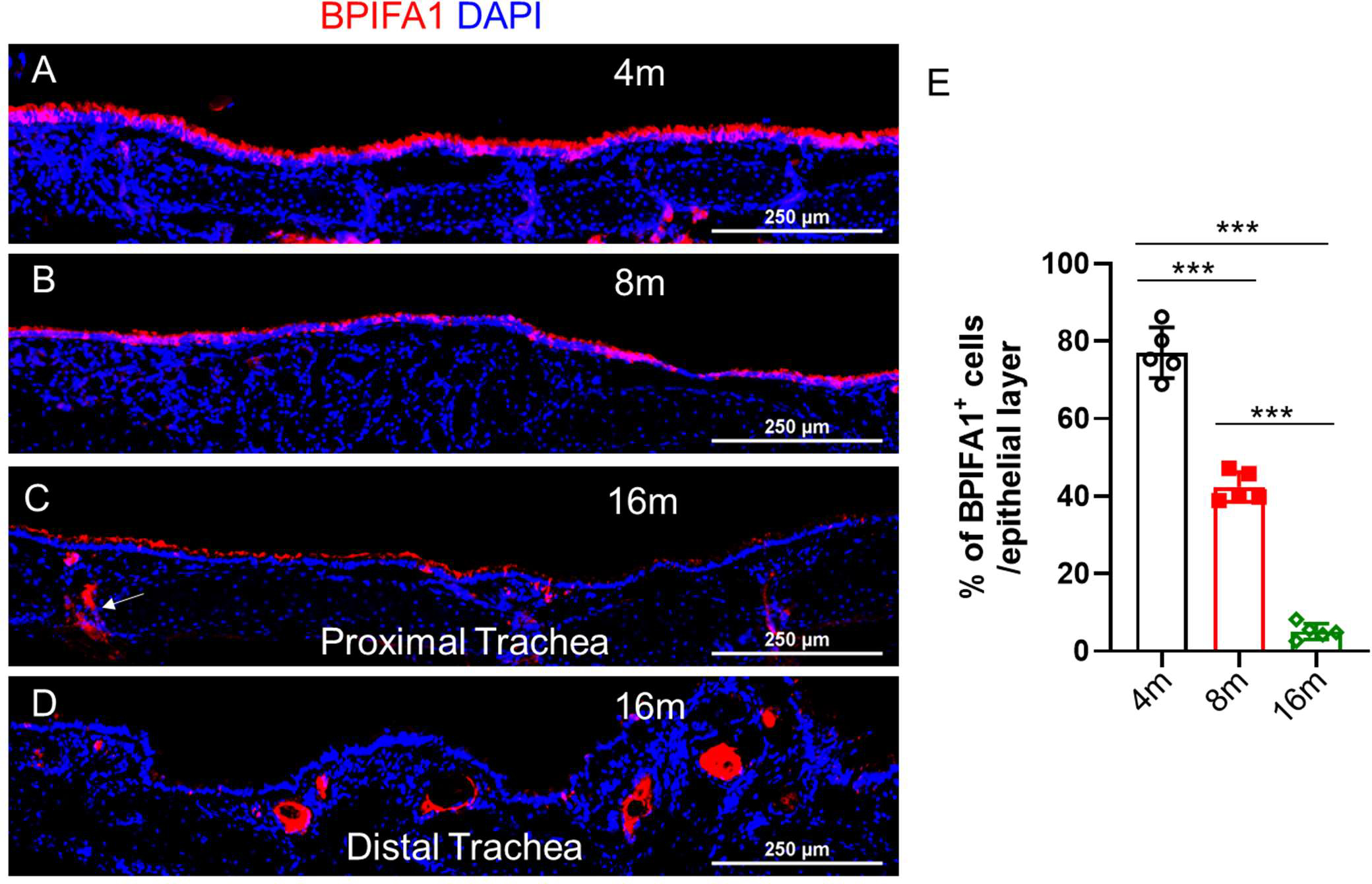
BPIFA1^+^ airway serous cells in the mouse trachea decrease in abundance with aging. (**A**–**D**). Representative staining of BPIFA1 on the tracheas of mice at 4 months (young), 8 months (pre-aged), and 16 months (aged). Scale bar: 250 μm. (**E**). Quantification of the percentage of BPIFA1 immuno-positive regions out of total tracheal epithelial layers at 4 months (young), 8 months (pre-aged), and 16 months (aged). mean ± SEM, *** *p* < 0.001. *n* = 5 independent tissues/sections.

Aging is accompanied by progressively declining mucosal immunity [21–24]. Aged mice are more susceptible to infection, exhibiting higher morbidity, faster weight loss, and slower recovery from infection [25]. Therefore, we examined whether aging impacts the distribution and abundance of airway serous cells. By staining for BPIFA1 in the tracheas of mice at 4 months (young), 8 months (pre-aged), and 16 months (aged), we observed a gradual reduction in the total number of airway serous cells with aging (Figure 4A–E). Interestingly, some BPIFA1-expressing serous cells were identified in the epithelial lining of submucosal collecting ducts in aged mouse tracheas (Figure 4C, indicated by an arrow). Additionally, we observed numerous gland-like structures in the distal trachea of aged mice, consistent with previous reports [26]. These gland-like structures, which do not have an opening to the surface lumen, were filled with secreted BPIFA1.

## 4. Discussion

Secretory cells located in airway glands are an important component of mucociliary clearance mechanisms in the normal lung, and alterations in the phenotype of these cells are associated with the pathogenesis of several lung diseases, including COPD and cystic fibrosis (CF). Various secretory cells contribute to ASL and are important contributors to pathogen clearance [18,27]. In large human airways, goblet cells of the surface epithelium, as well as serous and mucous cells of the glands, are the principal secretory cell types [28]. Secretory tubules of the submucosal glands consist of serous cells in the acini and proximal mucous cells [29]. The abundance of serous cells in human airway glands (serous to mucous cell volume ratio: 61%:39%) suggests that evolutionary pressures have favored the development and persistence of the serous cell type [30]. The abundance of serous cells provides practical advantages in maintaining the antimicrobial milieu. Serous cells are a critical component of host innate immunity, with enriched secretion of many defensive molecules, including antimicrobial, antioxidative, and antiprotease, to equip the host to battle environmental challenges. Despite the importance of serous cells and their critical functions, studies of airway serous remain limited. One of the limitations of studying serous cells is the lack of appropriate biomarkers. The classical known marker for serous cells is lysozyme. However, lysozyme has much broader expression patterns in many other cell types than being a specific serous cell marker. Alternatively, BPIFA1 could be a useful marker for serous cells because its staining is limited to serous cells in the submucosal glands. Interestingly, our results showed that not all serous cells in submucosal glands were positive for BPIFA1 staining. Thus, it will be worth investigating if the BPIFA1-positive serous cells represent maturational or functional significance of serous cell sub-populations.

BPIFA1 is an abundantly secreted airway protein. We estimated that the concentration of BPIFA1 in nasal fluid ranged from 30~150 μg/mL as measured by semiquantitative Western analysis and our established double sandwiched ELISA method. This amount of BPIFA1 is roughly 2 to 3 orders (300–1200 folds) higher than reported β-defensin 2 (HBD-2) concentrations in the same biological fluid. The reported concentration of BPIFA1 in secretions obtained from human tracheal aspirates is even higher than observed from nasal lavage [15,16,31,32]. A fascinating question is why and how surface epithelial cells express BPIFA1. Serous cells are prevalent on the surface epithelium of pathogen-free rodents [33], in animals lacking submucosal glands [34,35], in the human fetus, and pathological lung diseases such as CF and COPD. Our results indicated that the BPIFA1-positive serous cells on the surface epithelium were different among species. Mouse tracheal and bronchial airways have extensive staining of BPIFA1, while healthy human surface epithelium shows sparse BPIFA1-positive cells and no observed BPIFA1 staining in pig airway epithelium. Mouse secretory cells on the surface epithelium contain phenotypic characteristics of glandular (serous and mucous) cells and express the glandular secretory proteins. This suggests that anatomical distinctions in mice are functionally compensated through shifting secretory cells to serous cell phenotype. Additional studies to characterize the BPIFA1-positive serous cells in human airway epithelium with different pulmonary diseases are ongoing to investigate if the BPIFA1-positive cells are a dynamic reflection of airway epithelial cells’ status of pathophysiological conditions. The limitation of this study is not identifying the cell of origin of serous cells. Based on their distinct distribution pattern, we speculate that the serous cells in mice are likely derived from airway basal cells, while the BPIFA1-positive cells in pigs and humans originate from submucosal gland (SMG) myoepithelial progenitor cells. To confirm the origin and fate of these BPIFA1-positive serous cells, further studies are needed, including p63-lineage tracing and in vitro differentiation assays using isolated SMG myoepithelial progenitors from humans and pigs. This variation in cell origin may reflect the functional diversity among higher vertebrates.

In summary, our herein study provides crucial knowledge of serous cells and characterizes their expression patterns in surface epithelium and submucosal glands of different species. This information is indispensable to advance the knowledge and understanding of serous cells and their significance in pulmonary diseases.
